# The preparation and characterization of graphene oxide-multiwalled minocycline coatings on ultrafine-grained titanium implants for enhanced performance studies

**DOI:** 10.3389/froh.2025.1565325

**Published:** 2025-04-23

**Authors:** Ying Liu, Chenyang Niu, Minghui Chu, Mingda Liu, Yanxia Chi

**Affiliations:** ^1^ Stomatology Collage of Jiamusi University, Jiamusi, Heilongjiang, China; ^2^ Key Laboratory of Oral Biomedical Materials and Clinical Application, Jiamusi, Heilongjiang, China; ^3^ The Second Affiliated Hospital of Jiamusi University, Jiamusi, Heilongjiang, China

**Keywords:** graphene oxide, ultrafine-grained titanium, electrochemical deposition, minocycline, antibacterial, cytocompatibility

## Abstract

As the dental implant restoration technology is constantly applied and developed, implant fracture and related infections have emerged as significant factors threatening the long-term outcome of implants. Hence, this experiment intends to bestow the implant itself with anti-fracture and antibacterial capabilities by utilizing ultrafine-grained titanium, which possesses relatively superior mechanical properties, as the implant material and depositing a graphene oxide-minocycline composite coating on its surface. This is done to prevent implant fracture and the initial attachment of early microorganisms, and to strive to impede the colonization of late microorganisms and the formation of infectious biofilms, thereby achieving long-term stability of the implant. The graphene oxide-minocycline composite coating can be successfully fabricated on the surface of ultrafine-grained titanium via electrochemical deposition and liquid-phase deposition techniques, which can enhance the hydrophilicity of ultrafine-grained titanium and exhibit good coating adhesion. It demonstrates excellent antibacterial properties against *Staphylococcus aureus*, has no *in vitro* hemolysis, and shows no obvious cytotoxicity to mouse pre-osteoblasts.

## 1 Introduction

The fracture of artificial dental implants and microbial infections are the primary factors influencing the success of implants. Literature reports indicate that grain refinement can enhance the mechanical properties of metallic materials (1). The preparation of ultrafine-grained pure titanium materials through equal channel angular pressing (ECAP) treatment on pure titanium, without introducing other elements, can significantly improve the mechanical properties of pure titanium (2, 3). *In vitro* and *in vivo* experiments have verified that ultrafine-grained pure titanium can better facilitate osteoblast adhesion and proliferation and promote bone tissue growth. Hence, ultrafine-grained pure titanium is an ideal material for fabricating dental implants.

Early microbial colonization might occur during the surgical process of implant insertion or in the early stage of postoperative healing. The bacterial biofilm formed in the early stage of postoperative healing can prevent the epithelial closure on the implant surface, resulting in irreversible bacterial adhesion and proliferation, endangering the osseointegration process. Therefore, the inhibition of bacteria in the early stage is of great significance for the long-term success of the implant (4, 5). Late-stage microbial infections can trigger biological complications, such as inflammation of the soft and hard tissues around the implant and the disruption of the osseointegration that has already formed around the implant, causing the implant to loosen or even fall off. The clinical treatment plans for peri-implant infectious diseases remain undetermined, and the treatment effects are difficult to predict (6).

At present, significant progress has been made in the research on surface treatment technologies of implants. From traditional mechanical processing, acid etching treatment to novel nano-technology, bioactive coatings, and biomimetic surface treatment, the biocompatibility, bone integration ability, and antibacterial performance of implants have been continuously improved. However, there are few studies that focus on developing multifunctional surface treatment technologies to achieve the synergistic effects of multiple functions such as antibacterial, promoting bone integration, and anti-inflammatory (7–9). Due to the presence of a large number of oxygen-containing functional groups such as hydroxyl (-OH), epoxy (-0-), carboxyl (-COOH), and carbonyl (C = O) in graphene oxide, it has good dispersibility and hydrophilicity. Due to the sp2 hybridization of carbon atoms, graphene oxide has a high specific surface area and π-π conjugated structure. Therefore, graphene oxide can effectively load minocycline hydrochloride on the titanium surface. GO has excellent properties in terms of mechanical, electrical, and thermal properties, and the chemical bonds on the surface of GO contain many oxygen-containing functional group structures (10). These oxygen-containing groups of GO give it many properties such as dispersibility and hydrophilicity, and can also serve as activation sites to provide binding sites for the preparation of composite materials with other materials (11). Based on the above theories, this study intends to construct a graphene oxide (GO) coating on the surface of ultrafine-grained titanium implants through electrochemical deposition, and then load the antibacterial active coating of minocycline (MC) by liquid phase deposition technology, and conduct research on its physical characterization and biological performance. While ensuring good biological compatibility and osteogenic performance, it endows the implant surface with dual functions of contact antibacterial and drug release antibacterial, which can effectively inhibit the initial attachment of microorganisms in the early stage and prevent the colonization of late-stage microorganisms and the formation of infectious biofilms. This can prevent the occurrence of peri-implant infectious diseases and ensure the long-term stability and reliability of the implant.

## 2 Materials and methods

### 2.1 Materials and reagents

Ultrafine-grained pure titanium (Carpenter, USA), Aqueous solution of graphene oxide (Carbon Rich Graphene Technology, China), Minocycline hydrochloride powder (Meilin, China), Mouse embryonic osteoblasts (MC3T3-E1) Cell Bank of the Chinese Academy of Sciences, CCK-8 kit Biosharp Co., Ltd., Staphylococcus aureus (BNCC 186335), Phalloidin labeling solution (Qianchen Company, China).

### 2.2 Equipment and instruments

Scanning electron microscope (SEM) (JSM-6360LV model, JEOL, Japan), Energy dispersive spectrum (EDS) analyzer (Oxford Instruments Ultim Max, UK), Contact angle meter (Zhongchen, China), Laser Raman spectrometer (SHIMADZU CORPORATION, Japan), Multifunctional microplate reader (Shanghai Maisha Biotechnology Co., Ltd.), WS coating adhesion automatic scratch tester (Jiamusi University), Enzyme-linked immunosorbent assay (ELISA) reader (Shanghai Maisha Biotechnology Co., Ltd.), Laser confocal microscope (OLYMPUS Corporation, Japan).

### 2.3 Sample preparation

The surface of the ultrafine-grained titanium sheet was polished to be smooth and free of any marks. It was subsequently subjected to sandblasting, acid etching, rinsing, and drying for subsequent use. The ultrafine-grained titanium sheet was employed as the anode, while graphite paper served as the cathode, and both were placed in a GO water dispersion solution (100 μg/ml) for electrophoretic deposition (voltage: 20 V, duration: 5 min). The ultrafine-grained titanium sheet was retrieved, rinsed with deionized water, and dried at 37°C in the room for 24 h, labeled as UFG-GO, serving as the experimental control group. The ultrafine-grained titanium sheet was labeled as UFG-TI and served as the blank control group. Minocycline was prepared in distilled water to form solutions with concentrations of 0.05 mg/ml, 0.1 mg/ml, 0.2 mg/ml, 0.4 mg/ml, and 0.8 mg/ml. Subsequently, each group of suspended specimens (previously treated with surface sandblasting and acid etching to form graphene oxide film layers) were completely immersed in different concentrations of minocycline solutions for 24 h at room temperature and in a dark environment. The specimens without grafted MC were rinsed with distilled water and air-dried in the dark at room temperature, labeled as UFG-GO-MC, serving as the experimental group.

### 2.4 Materials characterization and performance testing

The surface morphology and pore size of the coating were observed using a scanning electron microscope (SEM). The elemental distribution on the material surface was analyzed using an energy dispersive spectrometer (EDS). Raman spectroscopy was utilized to detect characteristic peaks (G peak and D peak), primarily for testing GO in the coating. The hydrophilic property of the coating was examined using a water contact angle (WCA). The adhesion of the coating on the prepared ultrafine-grained titanium surface was tested using a scratch tester.

### 2.5 Antibacterial performance testing

The antibacterial performance of the coating was evaluated through the inhibition zone method (ZOI) and the plate coating method. Three parallel samples were set for the UFG-GO group and each concentration gradient experimental group. The bacterial strain utilized in the experiment was *Staphylococcus aureus (S. aureus).*

### 2.6 Hemolysis experiment

The experimental animals selected were adult male rabbits that had undergone blood collection for examination and whose single blood draw was more than 2 weeks ago. They were placed in a dedicated fixator with their heads extended to the fullest extent. The ear margin hair was shaved off, and they were disinfected with alcohol. The ears were then heated at 40°C for 10 min to dilate the blood vessels. The ear vein was punctured with a syringe, and the blood flowed into the pre-vacuum EDTA-K₂ anticoagulant collection system (5 ml) by itself. The blood was thoroughly mixed by the inversion method. Note: This blood collection operation complies with the “Guidelines for the Welfare and Ethics Review of Laboratory Animals” (GB/T 35892-2018) of China and the international animal welfare standards (such as the “Reduce, Optimize, Replace” principle of NC3Rs).

After ultraviolet sterilization of each coating group specimen, the experimental extract solution was prepared at a ratio of the specimen surface area to the volume of the physiological saline extract medium of 2:1 (cm²/ml). Then, 0.2 ml of anticoagulant diluted rabbit blood was added to 5 ml of each experimental group, 5 ml of the positive control group, and 5 ml of the negative control group. Three parallel controls were established for each group. The samples were centrifuged for 5 min (2,000 r/min), and 200 μl of the supernatant was taken from each group of centrifuge tubes and placed in a 96-well microplate. The absorbance value at 450 nm was detected using a microplate reader, and the hemolysis rate was calculated to assess the hemolytic performance.

### 2.7 Cell experiment

The single-cell suspension of mouse pre-osteoblast cells (MC3T3-E1) was inoculated on the surface of the 5 groups of specimens and incubated for 30 min. After the cells achieved initial adhesion, the culture medium was added for continued incubation. After 1 day, 3 days, and 5 days, the specimens were retrieved and co-cultured with the CCK-8 reaction solution for 2 h. The absorbance value at 450 nm was detected using a multi-functional microplate reader.

The third-generation MC3T3-E1 osteoblast cells in the logarithmic growth phase were cultured with the blank control group, the experimental control group, and the experimental group (MC concentration of 0.1 mg/ml) for 24 h The cells were stained with green fluorescence and observed under a laser confocal microscope for the morphology of osteoblasts.

### 2.8 Statistical analysis

Three parallel samples were established for each experiment. Graphpad Prism 9.5 software was utilized to process the data. All data underwent normal distribution tests before statistical analysis. All experimental data were expressed as mean ± standard deviation (x ± s). The comparison of data means was conducted using one-way analysis of variance (One-way ANOVA), and a *P* value < 0.05 was regarded as statistically significant.

## 3 Results

**Figure 1:**
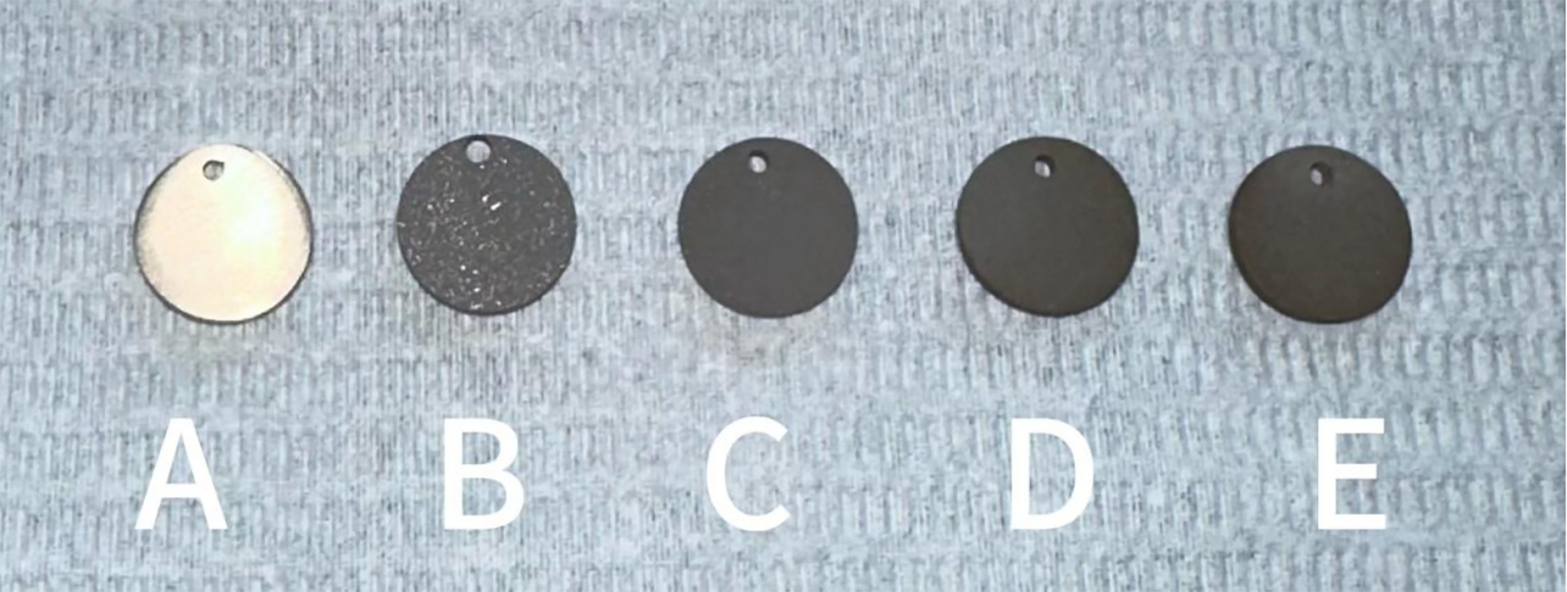
The surface morphology of each group of specimens. **(A)** The surface of the specimen after grinding can be seen with the naked eye to be shiny; **(B)** After sandblasting treatment, the surface of the material presents a matte color and has a rough texture; **(C)** After acid etching treatment, the surface of the material presents a uniform frosted texture; **(D)** After depositing GO coating, the surface color of the material deepens to dark brown; **(E)** After depositing GO-MC coating, the surface color of the material deepens to yellowish-brown.

**Figure 2:**
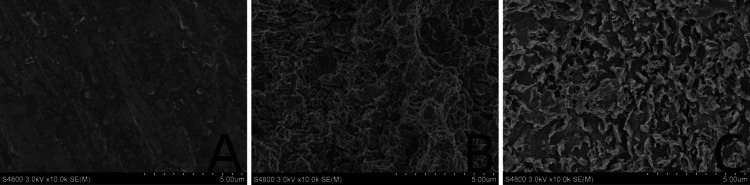
Surface topography map. **(A)** Smooth titanium sheet. **(B)** After sandblasting and acid etching. **(C)** After deposition with GO-MC.

**Figure 3:**
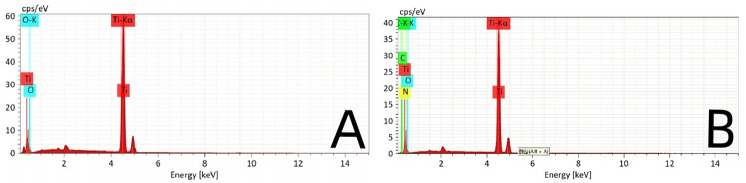
The EDS of the control group **(A)** and the experimental group **(B)**.

**Figure 4:**
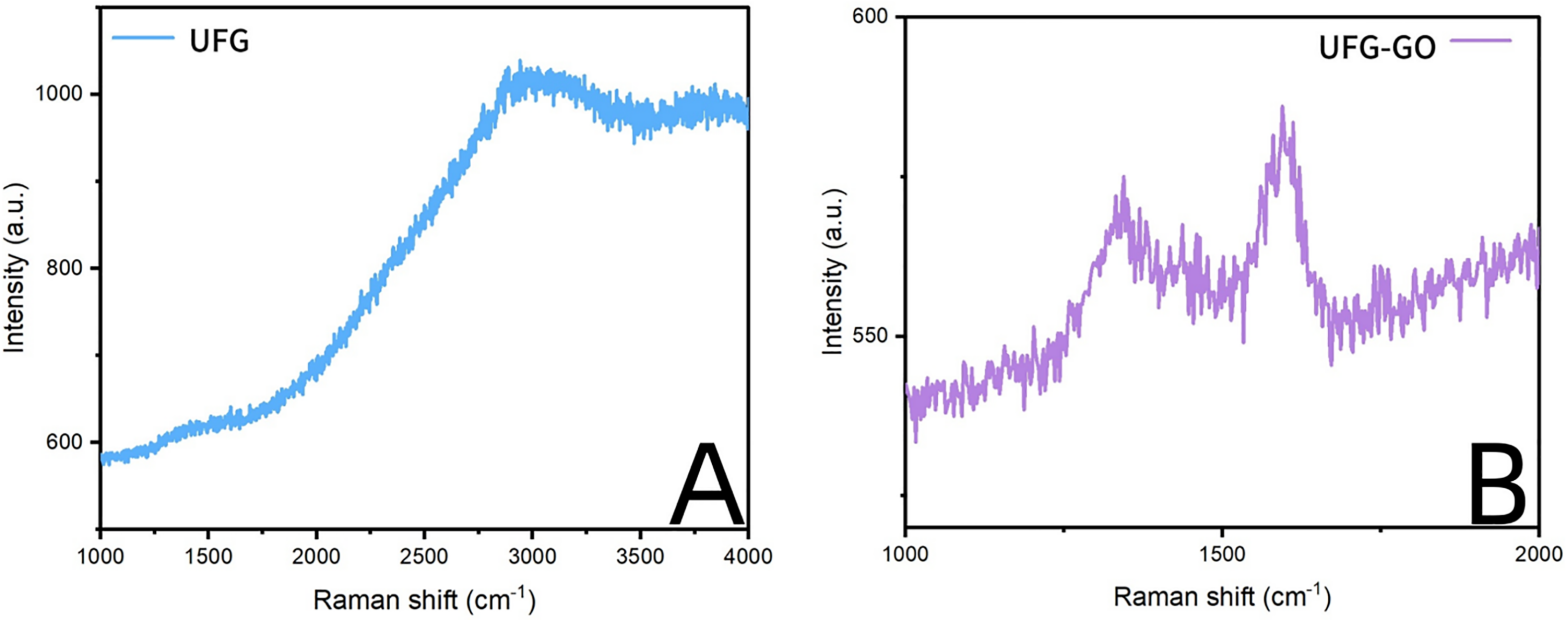
The Raman shift of the control group **(A)** and the experimental group **(B)**.

**Figure 5:**
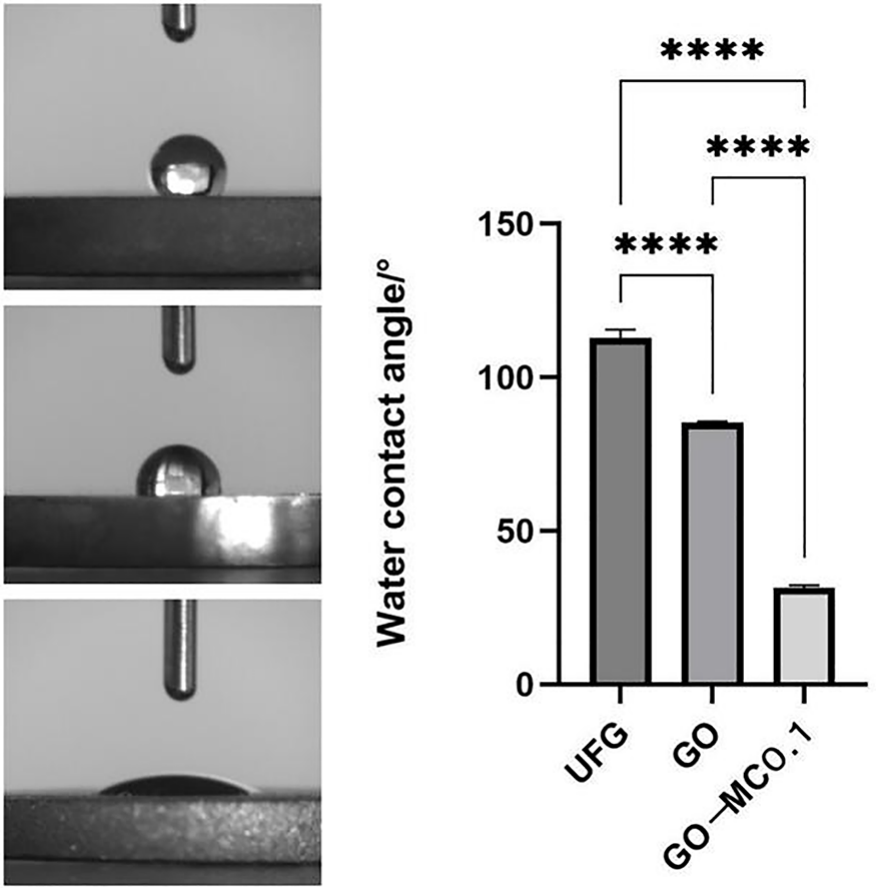
Contact angle of water.

**Figure 6:**
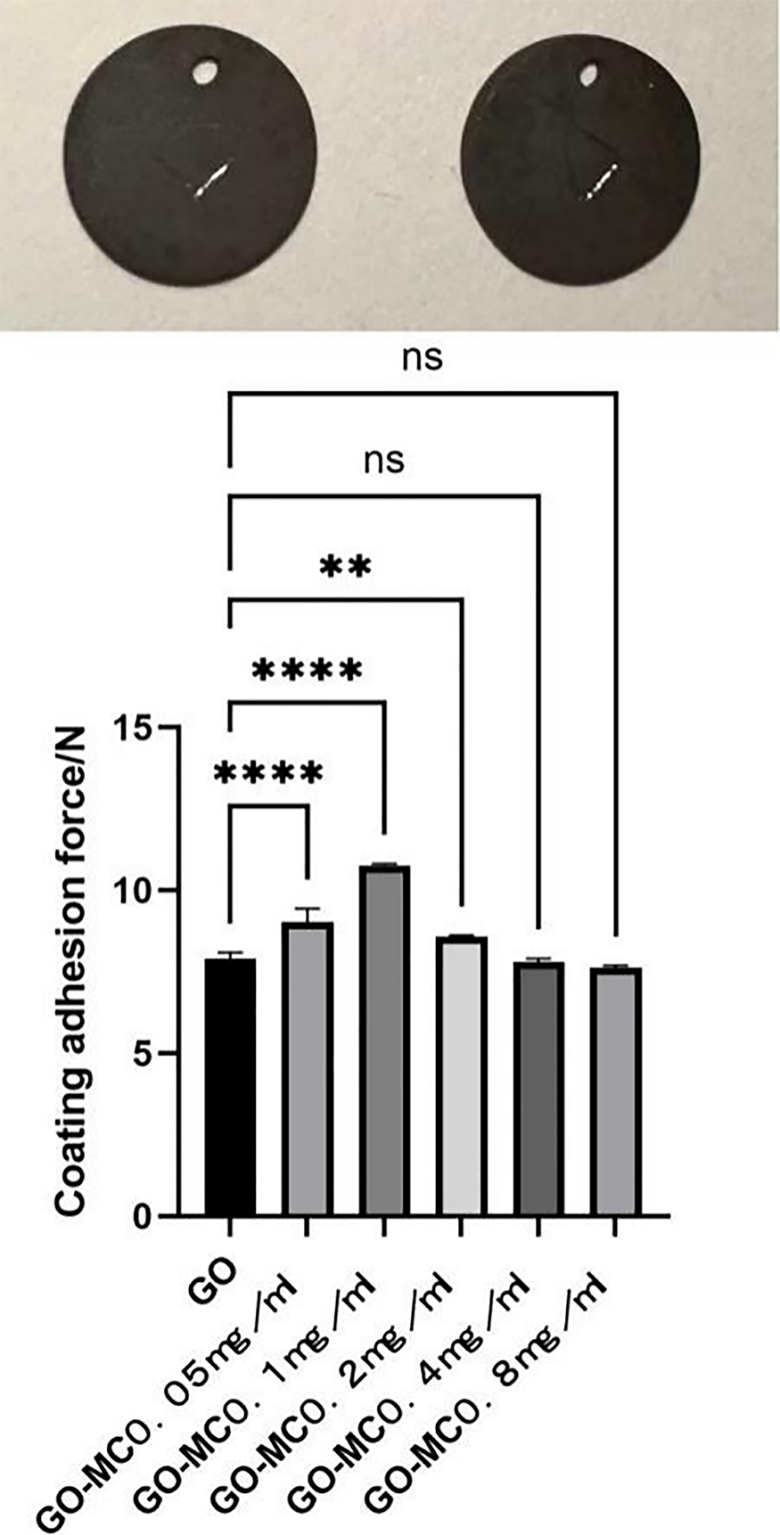
Scratch experiment. The picture shows a 5 mm scratch on the surface of the specimen, and the corresponding values are shown in the picture.

The surface of the ultrafine-grained titanium sheet exhibited a silver-gray metallic luster. After sandblasting, the surface appeared matte and rough. After acid etching, the material surface presented a uniform frosted texture. After depositing the GO coating, a tan film formed on the material surface. After depositing the GO-MC coating, the surface color of the material deepened to a yellowish-brown, as depicted in Figure 1 (from left to right: smooth ultrafine-grained titanium sheet, ultrafine-grained titanium sheet after sandblasting, ultrafine-grained titanium sheet after acid etching, ultrafine-grained titanium sheet after depositing GO coating, ultrafine-grained titanium sheet after depositing MC coating). As shown in Figure 2 from left to right, under the electron microscope, the surface of the smooth UFG-TI group displayed uniformly ground scratches; after sandblasting and acid etching, small depressions and secondary pores emerged; on the surface of the UFG-GO-MC group samples, a uniform film-like substance was observed covering the depressed morphology of the substrate, along with stacked wrinkles and flocculent structures. The contents of C and O in the UFG-GO group (right image) were increased compared to the UFG-TI group (left image); on the basis of the GO coating, the UFH-GO-MC group presented a peak of the characteristic element N of MC (Figure 3). The specimens after GO deposition all demonstrated the typical Raman spectra of graphene oxide, namely the D peak around 1,350 cm-1 and the G peak around 1,580 cm-1 (Figure 4). The contact angles of the smooth UFG-TI group, UFG-GO group, and UFG-GO-MC group (taking the 0.1 mg/ml concentration group as an example) were 113.02° ± 4.09°, 85.67° ± 0.26°, and 30.82° ± 1.67°, respectively, and the differences were statistically significant (*P* < 0.001) (Figures 5). The specimens of each group were examined by a universal testing machine scratch tester. The results indicated that the coating adhesion effect was the most favorable when the MC concentration was 0.2 mg/ml. As the concentration continued to increase, the coating adhesion effect deteriorated (Figure 6).

### 3.1 Antibacterial performance

#### 3.1.1 Results of plate coating experiments for different samples

**Figure 7:**
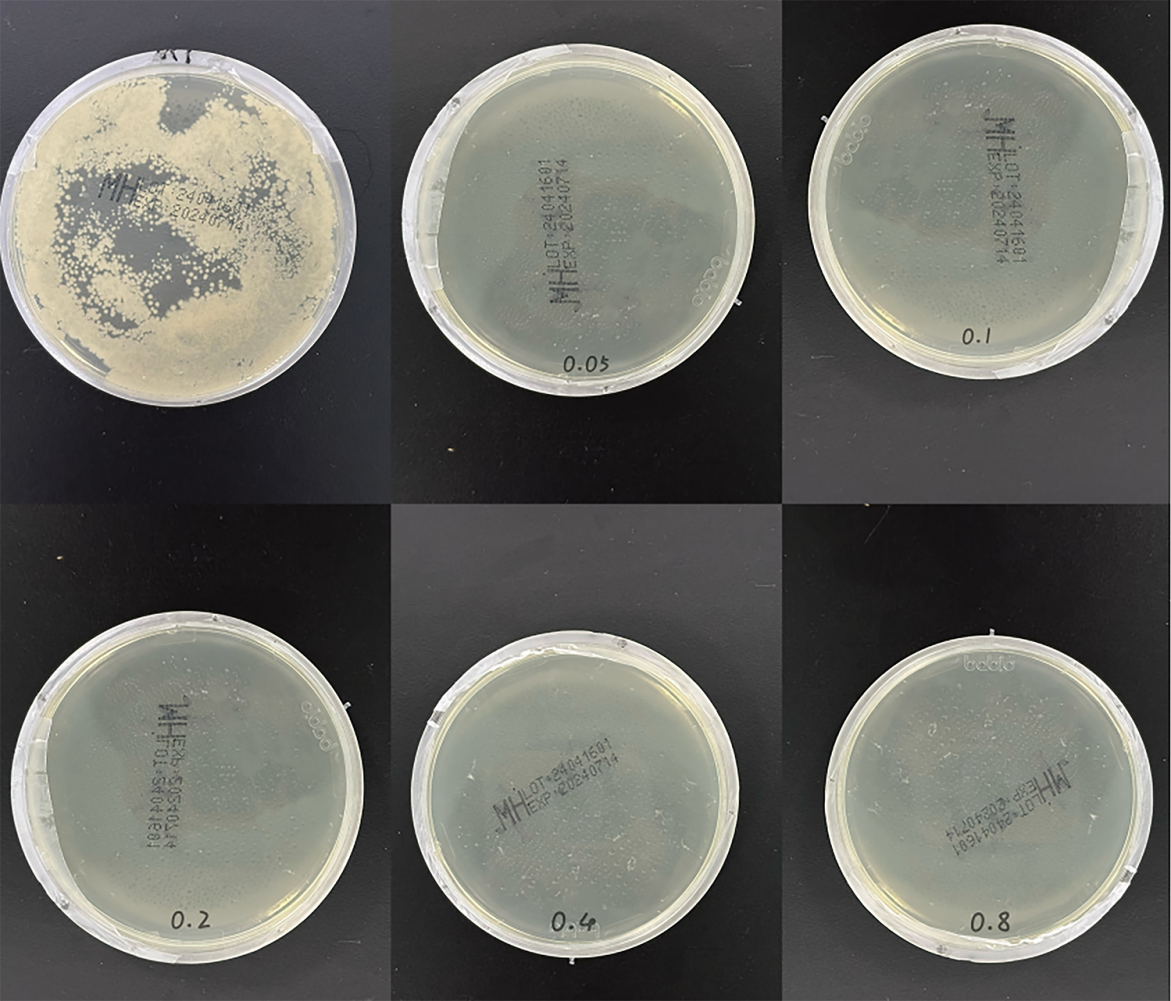
Plate coating experiment. The upper left figure shows the growth condition of Staphylococcus aureus in the control group (after 24-h culture), while the rest are the experimental results of each concentration group.

Figure 7 shows the co-culture of the extract solutions of each group of specimens with S. a. Obvious colony growth was observed on the plate of the control group. When the concentration of MC loaded on the plate was merely 0.05 mg/ml, good antibacterial properties were already manifested, with no distinct colonies observed. On the plates of the groups with concentrations of 0.1 mg/ml and above, almost no colony formation was detected on the surface.

#### 3.1.2 Results of the inhibition zone experiments for different samples

**Figure 8:**
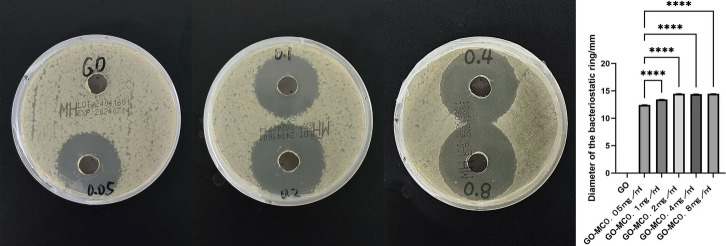
Bacteriostatic zone test. The left figure shows the inhibition zone diameters of Staphylococcus aureus in each group (after 24-h culture), while the right figure presents the data results of each group.

On the S. a. plate, only around the UFG-GO-MC group was a distinct inhibition zone observable. When the concentration of MC reached 0.2 mg/ml or above, the diameter of the inhibition zone did not exhibit a significant change with the increase in the concentration of MC and numerical statistics (Figure 8).

#### 3.1.3 Hemolysis experiment

**Figure 9:**
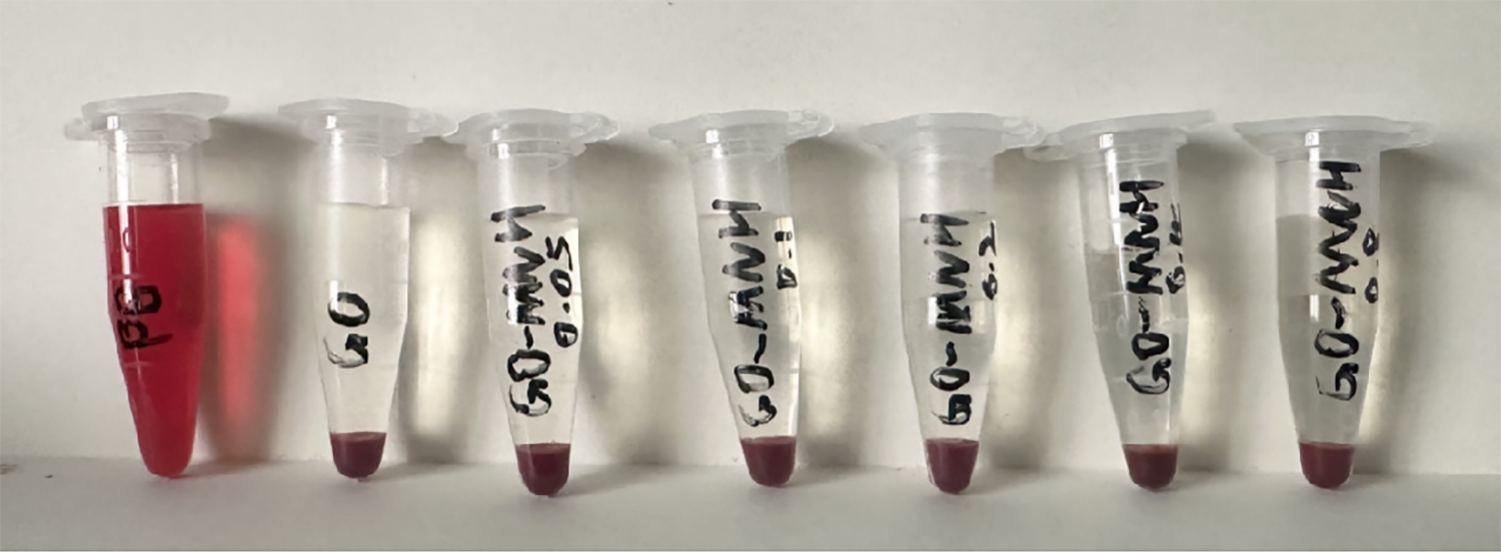
Observation of hemolysis experiment.

After centrifugation, a stratification phenomenon was clearly visible in each experimental group. The upper layer of the liquid was colorless and clear, while the lower layer showed red blood cell deposition. In the centrifuge tube of the positive control group, hemolysis was observed, and the entire tube was bright red (Figure 9).

The hemolysis rate = (OD of the experimental group—OD of the negative control group)/(OD of the positive control group—OD of the negative control group) × 100%.

According to the 5% hemolysis rate stipulated in ISO10993 “Biological Evaluation Standard for Medical Devices” as the evaluation criterion, an experimental hemolysis rate greater than 5% is considered positive and indicates hemolyticity; less than 5% is considered negative and indicates no hemolyticity.

The OD values of each group were measured using an enzyme-labeled immunosorbent assay reader. The hemolysis rate of the control group (normal saline) was 0.16%, and the hemolysis rates of the experimental groups with 0.05 mg/ml to 0.8 mg/ml GO-MC were 0.20%, 0.20%, 0.20%, 0.82%, and 0.41% respectively, all of which were less than the standard 5%. The results proved that the GO-MC composite film layers prepared on ultrafine-grained titanium through electrochemical deposition did not cause *in vitro* hemolysis.

#### 3.1.4 Cell experiment

CCK-8 experiments were performed on each group of specimens after 1, 3, and 5 days of cell culture, and the OD values of each group were detected using an enzyme marker. The results are presented in Table 1. The results of this experiment indicate that one day after inoculation, the OD values of the 0.05 mg/ml, 0.1 mg/ml, 0.2 mg/ml, 0.4 mg/ml, and 0.8 mg/ml UFG-GO-MC groups were all higher than those of the UFG-GO control group (*P* < 0.05), suggesting that the experimental groups facilitated cell proliferation. After the third day, the changes in OD values among the groups became increasingly evident. After 3 and 5 days, there were significant differences in the mean OD values between the UFG-GO control group and the 0.05 mg/ml, 0.1 mg/ml, and 0.2 mg/ml experimental groups (*P* < 0.05). Moreover, the absorbance value of the 0.1 mg/ml group was the highest among the experimental groups and exhibited a significant difference from the OD value of the Ca/*P* control group (*P* < 0.01), suggesting that the 0.1 mg/ml group was the optimal concentration group among the UFG-GO-MC experimental groups prepared by the electrochemical deposition method. The cell conditions in the UFG-GO-MC experimental groups were better than those in the UFG-GO control group, possibly due to the hydrophilic property of minocycline, which provided favorable survival conditions for cell adhesion and proliferation. However, as the concentration of MC increased, cell proliferation was inhibited.

**Table 1:** OD values of cells in the control group and each experimental group (at 1d, 3d, and 5d).

Group	1d	3d	5d
UFG-Ti	0.544 ± 0.221	1.141 ± 0.154	1.892 ± 0.051
UFG-Ti-GO	0.577 ± 0.335	1.169 ± 0.132	1.943 ± 0.023
0.05 mg/ml	0.622 ± 0.242	1.416 ± 0.215	2.113 ± 0.097
0.1 mg/ml	0.646 ± 0.252	1.456 ± 0.147	2.198 ± 0.063
0.2 mg/ml	0.633 ± 0.127	1.402 ± 0.151	2.213 ± 0.043
0.4 mg/ml	0.611 ± 0.387	1.356 ± 0.143	1.963 ± 0.086
0.8 mg/ml	0.601 ± 0.125	1.303 ± 0.171	1.875 ± 0.042

**Figure 10:**
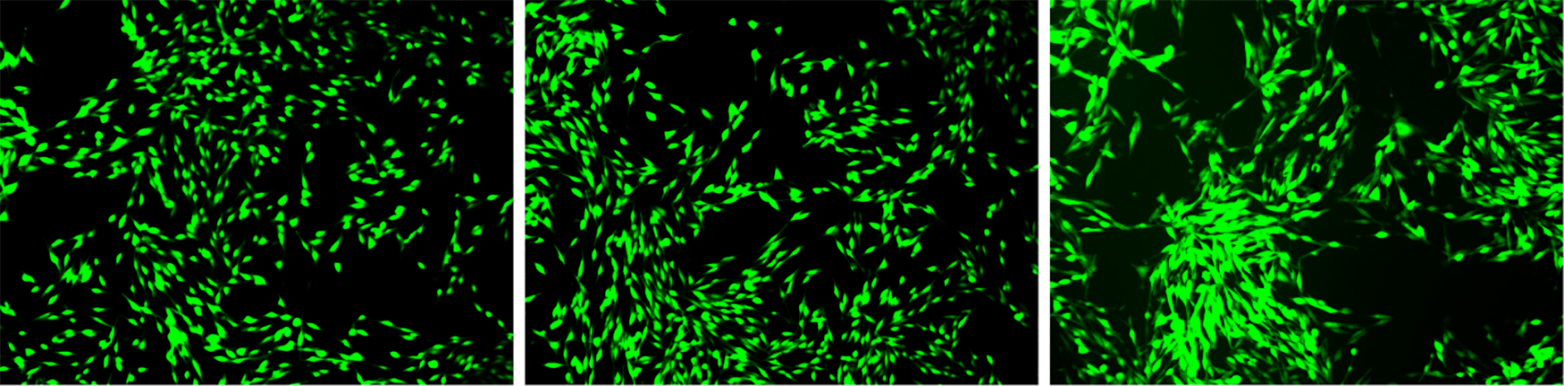
Observation of cell morphology by fluorescence staining. The growth conditions of each osteoblast cell (after 24 h of culture) are as follows: from left to right, it is the blank control group, the experimental control group, and the experimental group.

After 24 h of culturing MC3T3-E1 osteoblasts with each group, observations were made under a laser confocal microscope. Differences were noted in the number and morphology of cells between the experimental group and the control group. In the experimental group, finger-like protrusions and filopodia extended from the cell body, and the extension of filopodia was more complete compared to the control group. The intercellular relationships were closer and the cell count increased (as shown in Figure 10). This indicates that, at the cellular level, the graphene oxide-minocycline composite coating on the surface of the specimens can promote the adhesion and proliferation of MC3T3-E1.

## 4 Discussion

With the continuous maturation and application of implant restoration technology, an increasing number of studies have reported both short-term and long-term complications associated with oral implant restoration technology. Among them, implant fracture and peri-implantitis caused by bacterial infection are the most common and severe complications. Titanium and its alloys exhibit good corrosion resistance; however, their mechanical strength is low, and there is a risk of cracking and fracture under load pressure. Titanium is an inert metal and does not inherently possess antibacterial capabilities. Segal proposed that through equal channel angular pressing (ECAP), by applying shear deformation forces to perform severe plastic deformation (SPD) on materials, not only are the original characteristics of the materials retained, but also the yield strength and tensile strength of ultrafine-grained titanium are significantly enhanced compared to pure titanium (2). Studies have indicated that ultrafine-grained titanium prepared from pure titanium via SPD technology has uniform nanoscale grain size and a consistent structure. Due to the significant increase in grain interface density, the strength and toughness of pure titanium materials can be greatly enhanced (12, 13). The nanoscale micro-grain interface also renders the hydrophilicity of ultrafine-grained titanium higher than that of pure titanium (1). Some scholars have investigated the corrosion resistance of ultrafine-grained titanium and pure titanium using Ringer's solution and discovered that the corrosion resistance and anti-corrosion performance of ultrafine-grained titanium are superior to those of pure titanium (14). Park et al. demonstrated that ultrafine-grained titanium is more conducive to the growth of osteoblasts on its surface, possesses better osteoblast adhesion and proliferation capabilities, and exhibits superior biocompatibility (15). Future research on ultrafine-grained titanium should not only aim for higher ultimate and yield strengths but also approach the elastic modulus of the jawbone to achieve biomimetic properties of the material and bone (16).

The key to implant infection lies in the formation of biofilms, which commences immediately after bacteria adhere to the surface. By repelling or killing bacteria approaching the implant surface, the adhesion of live planktonic microorganisms in the environment can be prevented. A considerable number of studies have reported titanium surface modification and coating modification techniques aimed at enhancing the antibacterial properties of implants to minimize bacterial adhesion and colonization, inhibit biofilm formation, and provide effective bactericidal effects to improve the success rate of implants (17). Graphene oxide exhibits broad-spectrum antibacterial activity, and its mechanism mainly involves physical damage and oxidative stress (18). Physical damage includes edge-cutting effect and encapsulation effect (19). The edge-cutting effect refers to the sharp edges of graphene oxide that can directly damage the bacterial cell membrane, leading to leakage of cellular contents (20, 21). The encapsulation effect is that the large specific surface area of graphene oxide can encapsulate bacteria, blocking the material exchange between them and the external environment, thereby inhibiting bacterial growth. Oxidative stress includes the generation of reactive oxygen species (ROS) and electron transfer. Graphene oxide can induce the production of ROS (such as superoxide anion, hydroxyl radical, etc.), causing oxidative damage to bacterial DNA, proteins, and lipids. The conductivity of graphene oxide can interfere with the electron transfer chain of bacteria and affect their energy metabolism. In addition, the carboxyl, hydroxyl, and epoxy groups on the surface of graphene oxide can interact with the bacterial cell wall, destroying its structural integrity (22). Jin et al. all successfully prepared graphene oxide coatings on titanium sheets using electrophoretic deposition technology, with varying experimental conditions such as electrolyte concentration, reaction time, and applied voltage (23–25). Electrophoretic deposition technology is relatively straightforward, economical, and efficient, with a simple preparation process and no requirement for other coupling agents or chemical reagents, reducing interference with experimental bacteria and cells. In the future, the coating can be further optimized by adjusting the preparation parameters.

Staphylococcus aureus is highly prevalent in surgical incision infections (26). Staphylococcus aureus has a particular affinity for titanium and is the main pathogen of purulent peri-implantitis (27). Graphene oxide can cause irreversible damage to bacteria through mechanisms such as penetrating and puncturing the bacterial cell membrane, inducing mechanical stress by wrapping, extracting phospholipids from the bacterial cell membrane, and generating oxidative stress reactions (20, 21, 28). Minocycline is a semi-synthetic tetracycline antibiotic with multiple functions such as broad-spectrum antibacterial, anti-inflammatory, immunomodulatory and neuroprotective effects. Compared with traditional broad-spectrum antibiotics, minocycline has stronger antibacterial activity, higher concentration and longer duration of action (29). After entering the patient's body, it can inhibit Staphylococcus, Klebsiella and Escherichia coli, hinder the synthesis of bacterial proteins, help exert antibacterial and bactericidal effects, alleviate periodontal tissue damage (30), and at the same time can inhibit the activity of collagenase and various metalloproteinases, enhance affinity for bone tissue, inhibit the destruction of connective tissue and bone tissue, thereby facilitating periodontal tissue regeneration and improving therapeutic efficacy. In this study, it was found that the GO-MC coating demonstrated a favorable antibacterial effect after the combined application of the two, with dual antibacterial effects of contact killing and release killing. The results of the plate coating method and the inhibition zone experiment indicated that the GO coating had an antibacterial effect on directly contacted bacteria, but the antibacterial rate was relatively low. The GO-MC coating displayed good antibacterial effects in all experiments, not only significantly inhibiting the adhesion of bacteria on the material surface and the formation of biofilms on the material surface but also dissolving in the blood and body fluids around the implant, increasing the local concentration of MC and exerting an antibacterial effect on the surrounding environment. After GO successfully loaded MC, its antibacterial performance was enhanced, complementing the antibacterial effect of pure GO and effectively inhibiting the formation of bacterial biofilms. Previous studies have shown that although MC is released relatively rapidly within 8 h under GO loading conditions, it can reach a stable state and release slowly after 8 h (31).

The biocompatibility and cytotoxicity resulting from surface modification while endowing the implant with a functional surface cannot be disregarded. Under specific circumstances, graphene materials have good biocompatibility and can promote cell proliferation and osseointegration of implants (32). However, the morphology and size of different types of graphene materials have varying effects on cells, and the majority exhibit cytotoxicity (23, 33). Chen, et al. discovered that MC may alter the membrane potential, interfere with the synthesis of mitochondrial DNA-encoded proteins, and reduce caspase expression, thereby inhibiting cell apoptosis, promoting cell proliferation and migration (34). Only by choosing the appropriate synthesis method and dosage, etc., can a biologically safe effect be achieved. In this study, the ultrafine-grained titanium surface coating prepared had no *in vitro* hemolytic effect and facilitated the adhesion of MC3T3-E1 cells on the material surface, which was consistent with the results of the contact angle experiment indicating an increase in hydrophilicity. Additionally, the extract of the UFG-GO-MC coating material in this study did not significantly inhibit the proliferation or cause the death of MC3T3-E1 cells.

This experiment started from the selection of the base material. Considering that ultrafine-grained pure titanium has good biocompatibility, the reasons for this were identified as follows: Firstly, ultrafine-grained pure titanium has better hydrophilic properties; secondly, ultrafine-grained pure titanium has more abundant surface microstructures. However, due to these two factors, some scholars found that ultrafine-grained pure titanium can also promote the adhesion and aggregation of bacteria on its surface (35, 36). Therefore, while taking advantage of the excellent mechanical properties, surface properties and good cell biocompatibility of ultrafine-grained pure titanium, it is also necessary to consider endowing it with the ability to inhibit bacterial adhesion and aggregation; otherwise, it may trigger or aggravate peri-implantitis. Thus, this experiment aimed to treat the ultrafine-grained titanium material with surface treatment and deposit antibacterial layers, in order to ensure its osteogenic performance while endowing it with antibacterial ability, providing more possibilities for the prevention and treatment of peri-implantitis.

During the preliminary preparation of the ultrafine-grained titanium specimens in this experiment, sandblasting and acid etching methods were adopted. That is, the ultrafine-grained titanium sheets were polished and then impacted on the surface with high-speed abrasive particles (alumina) to increase the surface roughness, and then the surface was corroded by acid solution (a mixture of hydrochloric acid and sulfuric acid) to form micro-nano structures. The synergistic effect of these two methods can significantly improve the biocompatibility and bone integration ability of ultrafine-grained titanium materials. On the other hand, through acid etching and subsequent ultrasonic cleaning, the residual sandblasted powder on the specimen surface can be effectively removed, ensuring the smooth progress of subsequent experiments.

This experiment deposited graphene oxide (GO) on the surface of ultrafine-grained titanium by electrochemical deposition technology, which can significantly improve the surface properties of titanium materials, enhance their biocompatibility, antibacterial performance and bone integration ability. On the other hand, due to its special structure, graphene can be used as a carrier for the second coating in this experiment. Using the liquid-phase deposition method, minocycline was successfully grafted on the surface of ultrafine-grained titanium after the GO coating was deposited, achieving osteogenic and dual-bactericidal effects. Graphene oxide is a double-edged sword in biology. How to correctly control its concentration is the key to exerting its effect. This study controlled the concentration of the graphene oxide aqueous solution at 100 μg/ml through domestic and foreign related research and *in vitro* experiments, and then carried out electrochemical deposition to ensure its biological safety. To explore the optimal concentration of minocycline for osteogenic and bactericidal effects, this experiment divided into five concentration gradients for subsequent experiments and comparative studies.

The material characterization experiments of this experiment proved that the GO-MC coating was successfully deposited (for example, the Raman spectroscopy results showed the special G peak and D peak of GO, indicating that the GO deposition was successful; the EDS results showed the introduction of characteristic elements of GO and MC). The water contact angle experiment indicated that after grafting MC, the hydrophilic property of the material was greatly increased, providing a foundation for the subsequent osteogenic cell adhesion and proliferation, and also indicating the successful loading of MC. The coating adhesion force experiment indicated that when the MC concentration was 0.2 mg/ml, the coating adhesion effect was the best. As the concentration continued to increase, the coating adhesion effect deteriorated, possibly due to the influence of the increase in concentration on the thickness and membranous structure of the coating. The specific reasons are worthy of further exploration to find methods to enhance the adhesion force and cohesion of the coating.

GO can cause irreversible damage to bacteria through methods such as penetrating and piercing the bacterial cell membrane, inducing mechanical stress, extracting phospholipids from the bacterial cell membrane, and generating oxidative stress reactions. The bacterial experiment results showed that GO has a certain degree of direct contact bactericidal ability; when the loaded MC concentration was only 0.05 mg/ml, this coating already showed good antibacterial properties, and the best antibacterial effect was achieved when the MC concentration was 0.2 mg/ml in the antibacterial ring experiment, while as the concentration increased, the size of the antibacterial ring did not change. After GO was successfully loaded with MC, the antibacterial performance was enhanced, complementing the antibacterial effect of pure GO, and effectively inhibiting the formation of bacterial biofilms. The specific antibacterial effect still needs to be explored through relevant *in vivo* experiments.

Cell proliferation 1, 3, and 5 days' OD values of each group showed that after 1 day of culture, the OD values of the experimental group were all higher than those of the control group, and the experimental group was conducive to promoting cell proliferation. And with the increase of days, the cell activity increased, and the absorbance value of the group with MC concentration of 0.1 mg/ml in the experimental group was the highest, indicating that the prepared ultrafine-grained titanium-based 0.1 mg/ml GO-MC composite coating was the most suitable concentration group. The morphological observation of cells showed that after 24 h of cell culture, the overall number and condition of cells in the experimental group were better than those of the control group. This result indicated that the ultrafine-grained titanium-based GO-MC group was more conducive to cell adhesion and proliferation. The hemolysis experiment showed that the coatings prepared in this experiment had no *in vitro* hemolytic activity and had good biological safety.

Based on the above experimental results: The ultrafine-grained titanium-based GO-MC composite membrane layer has a significant inhibitory effect on Staphylococcus aureus, promotes the adhesion and proliferation of osteoblasts, and an appropriate MC concentration is more conducive to the growth of osteoblasts.

## 5 Conclusion

This experiment successfully deposited GO on ultrafine-grained titanium through electrochemical deposition and loaded MC on GO. The prepared UFG-Ti-GO-MC coating material has good antibacterial performance against Staphylococcus aureus, no *in vitro* cytotoxicity, and does not affect osteoblast proliferation. Nevertheless, this study still has some shortcomings. Due to the complexity of the human oral environment, the torque-induced wear during implant surgery, the corrosion formed in the body environment, and whether this material affects the osteogenic-related processes all require further research. Future studies should strive to simulate clinical conditions more closely and further translate these *in vitro* research results into *in vivo* clinical trials. It is expected that the experimental materials in this study will provide new material options for dental implant technology.

## Data Availability

The original contributions presented in the study are included in the article/Supplementary Material, further inquiries can be directed to the corresponding author.

## References

[1] Kim TN Balakrishnan A Lee BC Kim WS Dvorankova B Smetana K *In vitro* fibroblast response to ultra fine grained titanium produced by a severe plastic deformation process. J Mater Sci Mater Med. (2008) 19(2):553–7. 10.1007/s10856-007-3204-5 17619956

[2] Alt V Bitschnau A Böhner F Heerich KE Magesin E Sewing A Effects of gentamicin and gentamicin-rgd coatings on bone ingrowth and biocompatibility of cementless joint prostheses: an experimental study in rabbits. Acta Biomater. (2011) 7(3):1274–80. 10.1016/j.actbio.2010.11.012 21081183

[3] Kreuzeder M Abad MD Primorac MM Hosemann P Maier V Kiener D. Fabrication and thermo-mechanical behavior of ultra-fine porous copper. J Mater Sci. (2015) 50(2):634–43. 10.1007/s10853-014-8622-4 25540464 PMC4270432

[4] Jain SS Schramm STJ Siddiqui DA Huo W Palmer KL Wilson TG Jr. Effects of multiple implantations of titanium healing abutments: surface characteristics and microbial colonization. Dent Mater. (2020) 36(9):e279–e91. 10.1016/j.dental.2020.05.016 32591158 PMC7429256

[5] Sun J Liu X Lyu C Hu Y Zou D He YS Synergistic antibacterial effect of graphene-coated titanium loaded with levofloxacin. Colloids Surf B Biointerfaces. (2021) 208:112090. 10.1016/j.colsurfb.2021.112090 34507071

[6] Zhao P Wang Q Zhang P Zhou X Nie L Liang X Clinical efficacy of chlorhexidine as an adjunct to mechanical therapy of peri-implant disease: a systematic review and meta-analysis. J Oral Implantol. (2021) 47(1):78–87. 10.1563/aaid-joi-D-19-00213 32663270

[7] Minato K Katsuta Y Otsuka Y Katsuragi H Watanabe F. Effects of toothbrush abrasion on surface and antibacterial properties of hydroxyapatite-tryptophan complex with gray titania. Odontology. (2021) 109(4):819–27. 10.1007/s10266-021-00604-5 33837507

[8] Wennerberg A Albrektsson T Andersson B Krol JJ. A histomorphometric and removal torque study of screw-shaped titanium implants with three different surface topographies. Clin Oral Implants Res. (1995) 6(1):24–30. 10.1034/j.1600-0501.1995.060103.x 7669864

[9] Sul YT Johansson C Wennerberg A Cho LR Chang BS Albrektsson T. Optimum surface properties of oxidized implants for reinforcement of osseointegration: surface chemistry, oxide thickness, porosity, roughness, and crystal structure. Int J Oral Maxillofac Implants. (2005) 20(3):349–59. 15973946

[10] Oz Y Barras A Sanyal R Boukherroub R Szunerits S Sanyal A. Functionalization of reduced graphene oxide via thiol-maleimide “click” chemistry: facile fabrication of targeted drug delivery vehicles. ACS Appl Mater Interfaces. (2017) 9(39):34194–203. 10.1021/acsami.7b08433 28905618

[11] Bulbul E Aksakal B. Synthesizing and characterization of nano-graphene oxide-reinforced hydroxyapatite coatings on laser treated Ti6al4v surfaces. Acta Bioeng Biomech. (2017) 19(4):171–80. 29507435

[12] Jinlong L Tongxiang L Chen W Limin D. Effect of ultrafine grain on tensile behaviour and corrosion resistance of the duplex stainless steel. Mater Sci Eng C Mater Biol Appl. (2016) 62:558–63. 10.1016/j.msec.2016.02.008 26952459

[13] Dyakonov GS Zemtsova E Mironov S Semenova IP Valiev RZ Semiatin SL. An EBSD investigation of ultrafine-grain titanium for biomedical applications. Mater Sci Eng A. (2015) 648:305–10. 10.1016/j.msea.2015.09.080

[14] Dheda SS Kim YK Melnyk C Liu W Mohamed FA. Corrosion and *in vitro* biocompatibility properties of cryomilled-spark plasma sintered commercially pure titanium. J Mater Sci Mater Med. (2013) 24(5):1239–49. 10.1007/s10856-013-4889-2 23423650

[15] Park JW Kim YJ Park CH Lee DH Ko YG Jang JH Enhanced osteoblast response to an equal channel angular pressing-processed pure titanium substrate with microrough surface topography. Acta Biomater. (2009) 5(8):3272–80. 10.1016/j.actbio.2009.04.038 19426841

[16] Zhang D Qiu D Gibson MA Zheng Y Fraser HL StJohn DH Additive manufacturing of ultrafine-grained high-strength titanium alloys. Nature. (2019) 576(7785):91–5. 10.1038/s41586-019-1783-1 31802014

[17] Chouirfa H Bouloussa H Migonney V Falentin-Daudré C. Review of titanium surface modification techniques and coatings for antibacterial applications. Acta Biomater. (2019) 83:37–54. 10.1016/j.actbio.2018.10.036 30541702

[18] Wei W Zhu J Liu Y Chen L Zhu W Ji H Graphene oxide-silver-coated sulfonated polyetheretherketone (ag/go-speek): a broad-spectrum antibacterial artificial bone implants. ACS Appl Bio Mater. (2024) 7(6):3981–90. 10.1021/acsabm.4c00338 38781457

[19] Zhao R Kong W Sun M Yang Y Liu W Lv M Highly stable graphene-based nanocomposite (go-pei-ag) with broad-spectrum, long-term antimicrobial activity and antibiofilm effects. ACS Appl Mater Interfaces. (2018) 10(21):17617–29. 10.1021/acsami.8b03185 29767946

[20] Li J Wang G Zhu H Zhang M Zheng X Di Z Antibacterial activity of large-area monolayer graphene film manipulated by charge transfer. Sci Rep. (2014) 4:4359. 10.1038/srep04359 24619247 PMC3950808

[21] Akhavan O Ghaderi E. Toxicity of graphene and graphene oxide nanowalls against bacteria. ACS Nano. (2010) 4(10):5731–6. 10.1021/nn101390x 20925398

[22] Liu X Xu C Fu C Xia D Wang F Yin H Graphene oxide-sensitized surface plasmon resonance biosensor of porcine reproductive and respiratory syndrome virus. Molecules. (2022) 27(12):3942. 10.3390/molecules27123942 35745065 PMC9229610

[23] Jin J Zhang L Shi M Zhang Y Wang Q. Ti-Go-Ag nanocomposite: the effect of content level on the antimicrobial activity and cytotoxicity. Int J Nanomedicine. (2017) 12:4209–24. 10.2147/ijn.S134843 28652728 PMC5473600

[24] Qiu J Geng H Wang D Qian S Zhu H Qiao Y Layer-number dependent antibacterial and osteogenic behaviors of graphene oxide electrophoretic deposited on titanium. ACS Appl Mater Interfaces. (2017) 9(14):12253–63. 10.1021/acsami.7b00314 28345852

[25] Suo L Jiang N Wang Y Wang P Chen J Pei X The enhancement of osseointegration using a graphene oxide/chitosan/hydroxyapatite composite coating on titanium fabricated by electrophoretic deposition. J Biomed Mater Res B Appl Biomater. (2019) 107(3):635–45. 10.1002/jbm.b.34156 29802685

[26] Seidelman JL Mantyh CR Anderson DJ. Surgical site infection prevention: a review. JAMA. (2023) 329(3):244–52. 10.1001/jama.2022.24075 36648463

[27] Zhuang LF Watt RM Mattheos N Si MS Lai HC Lang NP. Periodontal and peri-implant microbiota in patients with healthy and inflamed periodontal and peri-implant tissues. Clin Oral Implants Res. (2016) 27(1):13–21. 10.1111/clr.12508 25399962

[28] Al-Jumaili A Alancherry S Bazaka K Jacob MV. Review on the antimicrobial properties of carbon nanostructures. Materials (Basel). (2017) 10(9):1066. 10.3390/ma10091066 28892011 PMC5615720

[29] Passarelli PC Netti A Lopez MA Giaquinto EF De Rosa G Aureli G Local/topical antibiotics for peri-implantitis treatment: a systematic review. Antibiotics (Basel). (2021) 10(11):1298. 10.3390/antibiotics10111298 34827236 PMC8615130

[30] Wu Y Gu C Tong X. Clinical efficacy of minocycline hydrochloride for the treatment of peri-implant disease: a systematic review with meta-analysis of randomized controlled trials. J Oral Implantol. (2023) 49(3):245–52. 10.1563/aaid-joi-D-22-00023 36796073

[31] Qian W Qiu J Su J Liu X. Minocycline hydrochloride loaded on titanium by graphene oxide: an excellent antibacterial platform with the synergistic effect of contact-killing and release-killing. Biomater Sci. (2018) 6(2):304–13. 10.1039/c7bm00931c 29184938

[32] Qian W Qiu J Liu X. Minocycline hydrochloride-loaded graphene oxide films on implant abutments for peri-implantitis treatment in beagle dogs. J Periodontol. (2020) 91(6):792–9. 10.1002/jper.19-0285 31782532

[33] Li Q Shen A Wang Z. Enhanced osteogenic differentiation of BMSCS and M2-phenotype polarization of macrophages on a titanium surface modified with graphene oxide for potential implant applications. RSC Adv. (2020) 10(28):16537–50. 10.1039/c9ra10563h 35498860 PMC9052948

[34] Chen M Ona VO Li M Ferrante RJ Fink KB Zhu S Minocycline inhibits caspase-1 and caspase-3 expression and delays mortality in a transgenic mouse model of Huntington disease. Nat Med. (2000) 6(7):797–801. 10.1038/77528 10888929

[35] Truong VK Rundell S Lapovok R Estrin Y Wang JY Berndt CC Effect of ultrafine-grained titanium surfaces on adhesion of bacteria. Appl Microbiol Biotechnol. (2009) 83(5):925–37. 10.1007/s00253-009-1944-5 19296098

[36] Truong VK Lapovok R Estrin YS Rundell S Wang JY Fluke CJ The influence of nano-scale surface roughness on bacterial adhesion to ultrafine-grained titanium. Biomaterials. (2010) 31(13):3674–83. 10.1016/j.biomaterials.2010.01.071 20163851

